# A new donors’ *CYP3A5* and recipients’ *CYP3A4* cluster predicting tacrolimus disposition, and new-onset hypertension in Chinese liver transplant patients

**DOI:** 10.18632/oncotarget.19606

**Published:** 2017-07-26

**Authors:** Yuan Liu, Tao Zhang, Xiaoqing Zhang, Ling Ye, Haitao Gu, Lin Zhong, Hongcheng Sun, Chenlong Song, Zhihai Peng, Junwei Fan

**Affiliations:** ^1^ Department of Hepatobiliary Pancreatic Surgery, Shanghai General Hospital, Shanghai Jiao Tong University School of Medicine, Shanghai, China; ^2^ Department of Pharmacy, Shanghai Pulmonary Hospital, Tongji University School of Medicine, Shanghai, China

**Keywords:** liver transplantation, CYP3A5, CYP3A4, new-onset hypertension

## Abstract

**Aim:**

The purpose of the current study was to investigate individualized therapy of tacrolimus (Tac), as well as complications after liver transplantation (LT) with the known genetic determinants and clinical factors.

**Methods:**

In this retrospective study, two cohorts (n=170) from the China Liver Transplant Registry (CLTR) database from July 2007 to March 2015 were included.

**Results:**

Both donors’ CYP3A5*3 and recipients’ CYP3A4*1G had a correlation with Tac pharmacokinetics at four weeks (all *P*<0.05), except recipients’ CYP3A4*1G nearly had an association at week 2 (*P*=0.055). The model of donors’ CYP3A5*3, recipients’ CYP3A4*1G, and total bilirubin (TBL), for the prediction of Tac disposition, was better than donors’ CYP3A5*3 only at week 1, 2, and 3 (*P*=0.010, *P*=0.007, and *P*=0.010, respectively), but not apparent at week 4 (*P*=0.297). Besides, when the *P* value was greater than or equal to 0.6685 after considering the false-positive rate R=10%, the patients were considered to have a faster metabolism, according to the mentioned model. Interestingly, we found that if more than or equal to two alleles A were present in the combination of donors’ CYP3A5*3 and recipients’ CYP3A4*1G genotype, there was a lower Tac C/D ration at week 1, 2, and 3 (*P*<0.001, *P*=0.001, and *P*<0.001), except at week 4 (*P*=0.082), and the probability of new-onset hypertension was lesser (*P*<0.001).

**Conclusions:**

These data provided a potential basis for a comprehensive approach to predicting the Tac dose requirement in individual patients and provided a strategy for the effective prevention, early diagnosis of new-onset hypertension in Chinese LT recipients.

## INTRODUCTION

Tacrolimus (Tac) is an immunosuppressant drug that belongs to the class of calcineurin inhibitors and has an important role in the prevention of allograft rejection in liver transplantation (LT) [1, 2]. It is characterized by a narrow therapeutic index and large interpatient variabilities in its pharmacokinetic and pharmacodynamic profiles, and it displays a wide range of potentially severe drug-related toxicities [3–6]. Regardless of these unfavorable characteristics, Tac is recognized as one of the most important immunosuppressants in solid-organ transplantation [7, 8].

*CYP3A5* is the main catalyst of Tac metabolism, as we known. The 6986A > G variant in intron 3 of *CYP3A5* (CYP3A5*3) (rs776746), is known as one of the most important single nucleotide polymorphisms (SNPs) in *CYP3A5* [9]. The CYP3A5*3 *AA* or *AG* genotype (i.e., patients expressing *CYP3A5* protein) have a substantially higher Tac clearance, resulting in markedly higher Tac dose requirements when compared with that of CYP3A5*3 *GG* genotype (i.e. patients not expressing *CYP3A5* protein) [10–12]. On the basis of these findings, it has been hypothesized that individualizing the initial Tac dosage based on *CYP3A5* genotype (i.e., 0.30 mg/kg/day for CYP3A5*3 *AA or AG* genotype carriers and 0.15 mg/kg/day for CYP3A5*3 *GG* genotype patients, instead of the standard 0.20 mg/kg/day for all patients) might help avoid underexposure and overexposure to Tac early after LT [13]. As underexposure to Tac is related with an increased risk of acute rejection [14] and overexposure is related with an increased risk of drug-related toxicities, such as new-onset hypertension and new-onset diabetes mellitus after LT [15–17], individualized dosing might improve the quality of life and clinical outcomes after transplantation. There was a prospective randomized study showed that more number of patients within the desired Tac target range early after transplantation and a faster achievement of Tac trough (C0) levels according to the *CYP3A5* genotype of the patient [18], but it is essential to realize some limitations that are focusing solely on the *CYP3A5* genotype [19, 20].

Many factors including both clinical [e.g. age, sex, hemoglobin (Hb), albumin, total bilirubin (TBL)] and genetics [e.g. *CYP3A5* and *CYP3A4* SNPs] have been identified to affect the pharmacokinetics of Tac [21–23]. Regarding the genetics, it is greatly recognized that the CYP3A5 *3 genotype has a noticeable effect on Tac pharmacokinetics, whereas research on the effect of *CYP3A4* SNPs is limited [24, 25].

The CYP3A4*1G allele (rs2242480), a novel G-to-A substitution at position 82266 in intron 10 has been identified in the Japanese population [19, 26]. CYP3A4*1G can increase the activity of *CYP3A4* enzyme [27, 28], and several studies indicate that this SNP is related to the pharmacokinetics of Tac [2, 29], as well as responsible for the interindividual differences in cyclosporine disposition [30, 31]. Hence, we advocated that the interindividual differences in Tac pharmacokinetics *in vivo* might also be partially owing to the interindividual differences in the CYP3A4*1G activity. Based on this frame, we investigated the relationship between the CYP3A5*3 and CYP3A4*1G genotypes in liver transplant donors and recipients, and on the pharmacokinetics of Tac, and on the complication of liver transplantation (e.g. new-onset diabetes and new-onset hypertension), considering the known clinical determinants of Tac disposition.

## RESULTS

### Clinical characteristics

The clinical characteristics for all population (n=170), training set (n =100) and validating set (n=70) were shown in Table 1. All patients were Chinese in this study and tested 4 weeks in both the training set and validating sets after LT. The average age of all patients was 47.9±9.5 years, and the average weight was 66.3±12.3 kg. The age of training set was younger than that of the validating set; however, this trend did not to be statistically significant. The majority causes of our transplant patients were hepatocellular carcinoma caused by hepatitis B virus.

**Table 1:** Patient demographic data

	All Population (n=170)	Training Set (n=100)	Validating Set (n=70)
Age (years)	47.9±9.5	46.8±9.0	49.0±10.0
Sex: female/male n (%)	35/135 (20.6/79.4)	17/83 (17.0/83.0)	18/52 (25.7/74.3)
Weight(kg)	66.3±12.3	68.0±11.5	65.0±10.6
Length(m)	1.70±0.58	1.72±0.49	1.70±0.61
BMI (kg/m^2^)	23.0±3.4	23.6±3.6	22.7±3.3
Underlying liver disease (n)			
Hepatitis B	141	83	58
Hepatitis C	2	1	1
Hepatocellular carcinoma	98	61	37
Others	17	10	7
Hemoglobin (g/l)	97.6±19.0	99.7±14.1	94.5±24.2
GPT (U/l)	47.3±91.3	101.5±59.8	61.9±117.3
TBL (umol/l)	48.8±51.5	54.8±54.1	40.2±47.0
Albumin (g/l)	36.5±7.4	38.0±3.1	36.4±8.7
Creatinine (mg/dl)	65.2±23.0	66.4±31.9	63.1±17.3
New-onset Diabetes (yes/no) (%)	32/101 24.1/95.9	21/62 25.3/74.7	11/39 24.5/75.5
New-onset Hypertension (yes/no) (%)	24/110 17.9/82.1	16/67 19.3/80.7	8/43 14.8/85.2
New-onset Hyperlipidemia (yes/no) (%)	53/97 35.3/74.7	31/61 33.7/66.3	22/36 34.9/65.1

Date was presented with mean value±standard deviation or count (percentage).

BMI: body mass index; GPT: glutamate pyruvate transaminase; TBL: total bilirubin.

### Genotyping

All alleles frequency was in Hardy–Weinberg equilibrium (*P*>0.05). There was remarkable linkage disequilibrium between the donors’ CYP3A5*3 and donors’ CYP3A4*1G (r^2^ = 0.494, D′ = 0.722), also between recipients’ CYP3A5*3 and recipients’ CYP3A4*1G (r^2^ = 0.535, D′ = 0.828). No difference between donors and recipients in the allele and genotype frequency of CYP3A5*3 and CYP3A4*1G was found (Table 2).

**Table 2:** Genotype and allele frequency of CYP3A5*3 and CYP3A4*1G polymorphisms in liver transplant donors (*n*=100) and recipients (*n*=100) in the training set

Gene	SNP	Genotype frequency, % (n)			Allele frequency, % (n)	
*CYP3A4*	Rs2242480	GG	AG	AA	G	A
Donors #		0.51 (46)	0.41 (48)	0.08 (5)	0.71 (140)	0.29 (58)
Recipients		0.61 (60)	0.34 (37)	0.05 (3)	0.78 (157)	0.22 (43)
*CYP3A5*	Rs776746	GG	AG	AA	G	A
Donors #		0.49 (45)	0.42 (48)	0.09 (6)	0.70 (138)	0.30 (60)
Recipients		0.55 (53)	0.38 (42)	0.07 (5)	0.74 (148)	0.26 (52)

# One case with missing genotype.

### Effect of SNPs on Tac C/D ratios

The Tac C/D ratios at different time periods after LT were compared among patients with different donors’ and recipients’ CYP3A5*3 polymorphisms, as well as CYP3A4*1G polymorphisms in the training set (Table 3). There was a correlation of recipients’ CYP3A4*1G genotype with Tac C/D ratios at week 1, 3, and 4(*P* = 0.046, 0.015, and 0.024, respectively), and nearly at week 2(*P*=0.055). However, the association between donors’ *CYP3A4* *1G gene polymorphisms and Tac C/D ratios was not found. Contrary to CYP3A4 *1G genotype, donors’ CYP3A5*3 had a correlation with Tac pharmacokinetics at week 1, 2, 3, and 4(*P*< 0.001, *P* =0.032, *P* = 0.048, and *P* = 0.003, respectively), but for recipients’ CYP3A5*3, the association with Tac disposition was just found at week 1 and week 3 (*P* = 0.014 and *P* = 0.038 respectively).

**Table 3:** Comparison of Tac concentration/dose ratios in different groups of donors’ and recipients’ *CYP3A5* and *CYP3A4* polymorphisms at different times after drug initiation in the training set

Gene	Locus	Genotype	Week 1		Week 2		Week 3		Week 4	
			C/D ratios	*p*	C/D ratios	*p*	C/D ratios	*p*	C/D ratios	*p*
CYP3A4	Rs2242480	GG	466.83±77.65	0.121	178.82±27.40	0.898	160.91±43.83	0.278	200.81±29.43	0.074
Donors CYP3A4 Recipients	Rs2242480	AG+AA GG AG+AA	329.65±39.20 451.71±54.11 288.50±55.50	0.046	183.43±23.50 209.69±26.89 140.56±17.60	0.055	211.94±22.18 230.26±36.79 115.95±13.08	0.015	143.22±16.52 196.10±23.71 122.12±13.22	0.024
CYP3A5	Rs776746	GG	564.87±73.60	<0.001	201.43±26.60	0.032	234.57±42.81	0.048	218.48±26.45	0.003
Donors CYP3A5 Recipients	Rs776746	AG+AA GG AG+AA	252.07±31.63 477.05±58.52 280.41±49.02	0.014	164.44±23.80 205.45±25.67 151.44±23.23	0.131	142.22±22.20 223.96±37.11 133.23±21.66	0.038	125.75±17.08 189.51±24.16 139.54±18.21	0.123

Data was presented as mean±standard deviation. Comparison between groups was performed by t-test. *P*<0.05 was considered significant.

### Effect of combination SNPs on Tac C/D ratios

Donors’ CYP3A5*3 allele A and recipients’ CYP3A4*1G allele A were shown to be related to faster Tac metabolism as stated in Table 3. Hence, the allele A was further explored in a combination analysis in the training set. The associations between the number of alleles A with a fast metabolism and Tac C/D ratios were shown in Table 4. When the number of alleles A was greater than or equal to two, the patients were found to have lower Tac C/D ratios at week 1, 2, and 3 (*P* < 0.001, *P* =0.001, and *P* < 0.001, respectively), and closed to significant at week 4 (*P* = 0.082).

**Table 4:** Comparison of Tac concentration/dose(C/D) ratios in different groups of the numbers of allele A which is a combination of donors’ CYP3A5*3 and recipients’ CYP3A4*1G genotype in the training set

The number of allele A	N	Week 1		Week 2		Week 3		Week 4	
		C/D ratio	p	C/D ratio	p	C/D ratio	p	C/D ratio	p
<2	68	217.91±20.69	<0.001	110.66±14.88	0.001	155.58±26.92	<0.001	138.89±27.82	0.082
≥2	32	86.41±11.97		55.84±6.10		49.03±5.93		60.49±8.44	

The data was presented as mean±standard deviation. The comparison between groups was performed by Chi-square. *P*<0.05 was considered significant.

<2: GGGG, GGGA; ≥2: GGAA, GAAA, AAAA.

### The number of alleles A (combination of donors’ *CYP3A5* *3 and recipients’ CYP3A4*1G genotype) and TBL predicting Tac disposition: multivariate linear regression analysis

Table 5 showed the multivariate linear regression models predicting Tac daily dose requirements, Tac C0 level, dose-corrected Tac C0 level at week 1, 2, 3, and 4 in the training set. The incorporated cofactors included Hb, the number of allele A (combination of donors’ *CYP3A5* *3 and recipients’ CYP3A4*1G genotype), TBL as well as glutamic oxalacetic transaminase. All these factors had been reported to have potential effects on Tac pharmacokinetics and entered into the validating set in the multivariate linear regression analysis (Table 6), which showed that TBL was the second explanatory variable to be retained after the number of allele A, in the list of Tac pharmacokinetic parameters.

**Table 5:** Multivariate regression analysis in the training set

Dependent/explanatory variable	Parameter estimate	Adjust R^2^	P/P
Tacrolimus dose (mg/day)		0.332	<0.001
The number of allele A	0.456		<0.001
Hb	0.274		0.005
TBL	-0.180		0.063
Tacrolimus C0 (ng/ml)		0.180	<0.001
The number of allele A	-0.403		<0.001
GPT	0.162		0.080
Tacrolimus C0/dose (ng/ml/kg) (first week)		0.266	<0.001
The number of allele A	-0.458		<0.001
Diabetes	0.322		0.003
Tacrolimus C0/dose (ng/ml/kg) (second week)		0.447	<0.001
The number of allele A	-0.227		0.007
TBL	0.637		<0.001
Tacrolimus C0/dose (ng/ml/kg) (third week)		0.131	0.001
The number of allele A	-0.310		0.003
TBL	0.239		0.022
Tacrolimus C0/dose (ng/ml/kg) (fourth week)		0.314	<0.001
The number of allele A	-0.369		<0.001
TBL	0.306		0.003
Hb	-0.192		0.057
Age	-0.206		0.035

Data was performed by multivariate linear regression analysis. *P*<0.05 was considered significant. The number of allele A: combination of donors’ *CYP3A5* and recipients’ *CYP3A4* genotype; Hb: hemoglobin; GPT: glutamate pyruvate transaminase; TBL: total bilirubin.

**Table 6:** Multivariate regression analysis in the validating set

Dependent/explanatory variable	Parameter estimate	Adjust R^2^	P/p
Tacrolimus dose (mg/day)		0.206	<0.001
The number of allele A	0.395		0.001
TBL	-0.197		0.077
Tacrolimus C0(ng/ml)		0.207	<0.001
The number of allele A	-0.355		0.001
TBL	0.221		0.050
Age	-0.186		0.095
Tacrolimus C0/dose (ng/ml/kg) (First week)		0.333	<0.001
The number of allele A	-0.400		<0.001
TBL	0.363		0.001
Tacrolimus C0/dose (ng/ml/kg) (Second week)		0.261	<0.001
The number of allele A	-0.164		0.131
TBL	0.473		<0.002
Tacrolimus C0/dose (ng/ml/kg) (Third week)		0.098	0.015
The number of allele A	-0.281		0.023
TBL	0.163		0.183
Tacrolimus C0/dose (ng/ml/kg) (Forth week)		0.078	0.018
The number of allele A	-0.306	0.018	

Data was performed by multivariate linear regression analysis. *P*<0.05 was considered significant. The number of allele A: combination of donors’ CYP3A5*3 and recipients’ CYP3A4*1G genotype; TBL: total bilirubin.

### Donors’ *CYP3A5* genotype, recipients’ *CYP3A4* genotype, and TBL predicting the daily Tac dose corrected by weight: receiver operating characteristic (ROC) curve

**Figure 1:**
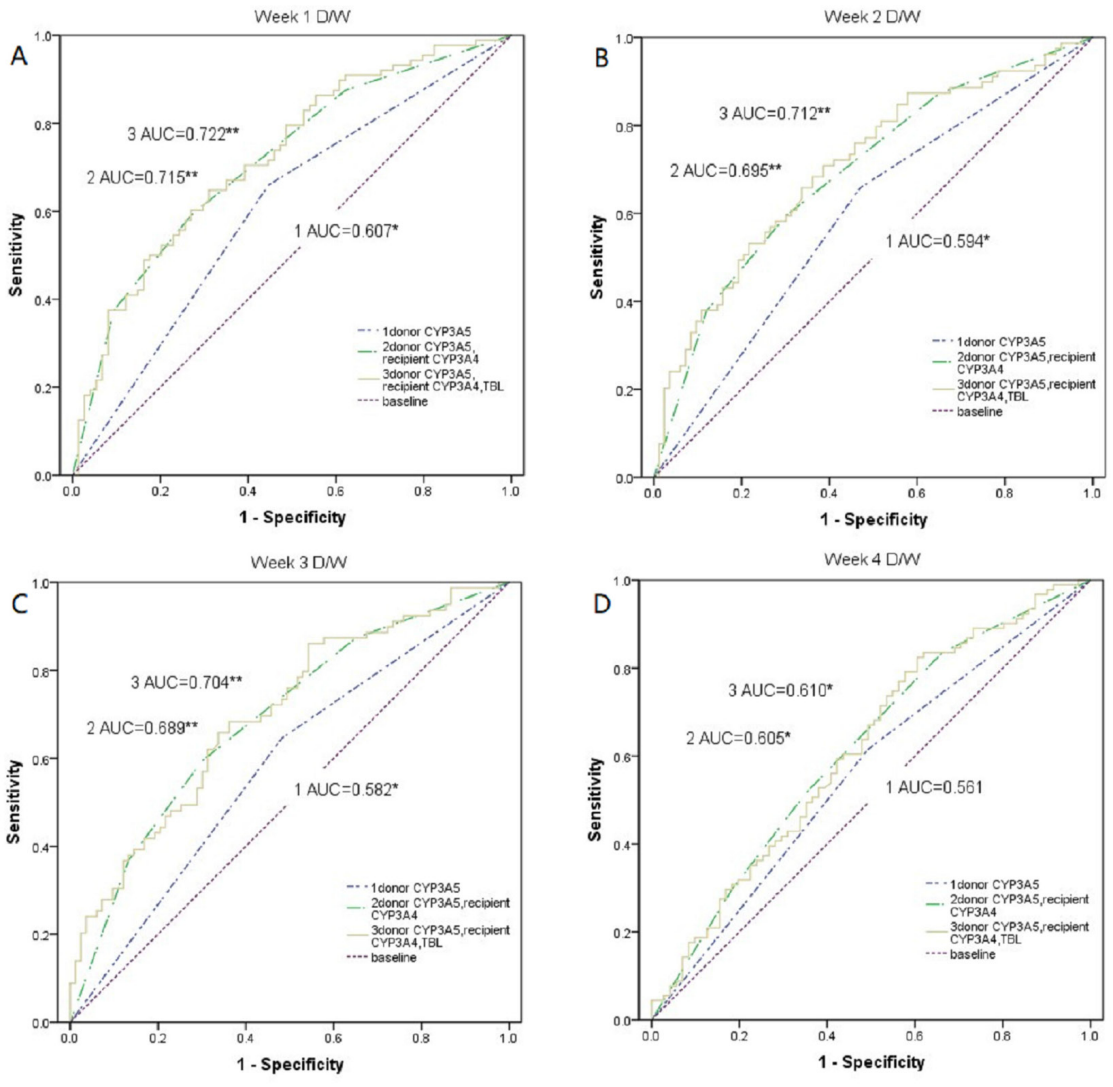
Comparison of weight-corrected Tac dose among the model of donors’ CYP3A5 *3, recipients’ CYP3A4*1G, and TBL, the model of donors’ CYP3A5 *3 and recipients’ CYP3A4*1G, and the model of donors’ CYP3A5 *3 alone **(A-D)** The weight-corrected Tac dose at 1–4 weeks after transplant were compared among the model of donors’ CYP3A5*3, recipients’ CYP3A4*1G, and TBL, the model of donors’ CYP3A5*3 and recipients’ CYP3A4*1G, and the model of donors’ CYP3A5*3 alone by using a receiver operating characteristic (ROC) curve. D/W, weight-corrected Tac dose; AUC, area under the curve; *, p < 0.05; **, p < 0.01

As shown in Figure 1, the model of donors’ *CYP3A5* genotype, recipients’ *CYP3A4* genotype, and TBL (*P*=0.009, *P*=0.010*, P*=0.007), and the model of donors’ *CYP3A5* and recipients’ *CYP3A4* genotype (*P*=0.013, *P*=0.021*, P*=0.014) had differences for predicting Tac disposition with the model of only CYP3A5 genotype at week 1, 2, and 3, while it was not observed at week 4(all *P*>0.05). Besides, no difference existed between the model of donors’ *CYP3A5* genotype, recipients’ *CYP3A4* genotype, and TBL and the model of donors’ *CYP3A5* and recipients’ *CYP3A4* genotype (all *P*>0.05). The area under the curve of the model for donors’ CYP3A5 genotype, recipients’ CYP3A4 genotype, and TBL was 0.722 (*P*=0.001, 95%CI:0.645-0.800), 0.712(*P*=0.001, 95%CI:0.636-0.780), 0.704(*P*=0.001, 95%CI:0.617-0.764), and 0.610(*P*=0.012, 95%CI:0.528-0.703) at week 1, 2, 3, and 4 respectively. As for the model of donors’ *CYP3A5* and recipients’ *CYP3A4* genotype, the corresponding area under the curve was 0.715(*P*=0.001, 95%CI:0.636-0.794), 0.695(*P*=0.001, 95%CI:0.614-0.776), 0.689(*P*=0.001, 95%CI:0.576-0.744), and 0.605(*P*=0.013, 95%CI:0.526-0.702), while the model of CYP3A5 genotype alone was 0.607(*P*=0.02, 95%CI:0.527-0.682), 0.594(*P*=0.039, 95%CI:0.514-0.617), 0.582(*P*>0.05, 95%CI:0.502-0.659), and 0.561(*P*>0.05, 95%CI:0.481-0.639). According to the data given in Table 7, diagnosis point of 0.6685 obtained after the false positive rate R=10% was taken. If the prediction probability value was greater than or equal to 0.6685 it was considered positive (i.e. diagnosed with a fast metabolism), whereas a value less than 0.6685 was diagnosed with a slow metabolism.

**Table 7:** Variable PRE_2 ROC curve coordinate point

Test Variable	Diagnosis point	Sensitivity	1-Specificity
PRE_2	0.0000000	1.000	1.000
	0.2399369	0.977	0.905
	0.2745092	0.955	0.811
	0.6493419	0.375	0.108
	0.6805440	0.375	0.095
	0.8349565	0.000	0.014
	1.0000000	0.000	0.000

Data was performed by ROC curve. ROC: recipient operative characteristic

PRE_2: donors’ CYP3A5*3, recipients’ CYP3A4*1G, and TBL.

### New-onset hypertension according to the combination of *CYP3A4* and *CYP3A5* polymorphisms

As shown in Table 8 that in our all study population, the association was observed between new-onset hypertension and the amounts of allele A (*P*=0.001), which the combination of donors’ *CYP3A5* *3 genotype and recipients’ CYP3A4*1G genotype. Besides, there was a difference between alleles A with greater than two and lesser than or equal to two. With increasing number of alleles associated with fast metabolism, the patients were found to have an increasingly low probability of the occurrence of hypertension. However, it was not apparent in the aspect of new-onset diabetes (*P*=0.637) and new-onset hyperlipidemia (*P*=0.941).

**Table 8:** Analysis of the number of allele A which is combination of donors’ CYP3A5*3 and recipients’ CYP3A4*1G genotype and complication in all population

The number of allele A	New-onset diabetes n (%)	p	New-onset hypertension n (%)	p	New-onset hyperlipidemia n (%)	p
≤2	29(91.7)	0.637	22(93.3)	<0.001	49(92.7)	0.941
>2	3(8.3)		2(6.7)		4(7.3)	

The data was presented with count (percentage). The comparison between groups was performed by Chi-square. *P*<0.05 was considered significant.

≤2: GGGG, GGGA, GGAA; >2: GAAA, AAAA.

## DISCUSSION AND CONCLUSIONS

It was well known that CYP3A5*3 played a crucial role in the metabolism of Tac disposition. Individuals with AA or AG genotype were *CYP3A5* expressors and metabolized Tac; however, those with the GG genotype barely metabolized Tac [10–12]. Our patients were mostly carriers of CYP3A5*3 GG and AG genotype; only 11 patients (donors and recipients) were carriers of AA genotype in the training set, and the predominant allele of CYP3A5*3 was *up to* 70–74%. In African–Americans, the CYP3A5*3 allele frequency was up to 55% compared with that observed in Caucasian subjects (85–95%) [9]. The data obtained in this study were between findings in American and Caucasian populations. Similarly, CYP3A4*1G allele frequency varied among different ethnic groups: 24.9% in Japanese and 22.1% in Chinese [19, 26]. In this study, the CYP3A4*1G 22–29% allele distribution in our patients was similar to that reported previously. In this context, compared to the liver, the role of the small intestine in the metabolism of drugs in receptor organisms could not be ignored. Since most clinical drugs delivers was oral, the role of the small intestine in drug absorption link was over-emphasized in the past, and the ability of absorption was underestimated. The expression of small intestinal *CYP3A4* was much higher than that of *CYP3A5*, accounting for 73% of the total *CYP3A* [33]. Thus, we were intrigued to examine the impact of combination with *CYP3A5* and *CYP3A4* polymorphisms on Tac pharmacokinetics in LT, considering the known clinical determinants.

First of all, our data indicated that donor *CYP3A5*- and recipient *CYP3A4*-mediated Tac metabolisms were both critical to Tac disposition *in vivo* [2]. This was consistent with the fact that *CYP3A4* existed mostly in the gastrointestinal tract of the recipient and *CYP3A5* presented mainly in the liver of the donor, both being sites of drug metabolism. Almost all studies had reported a lower Tac exposure and/or a higher dose requirement in individuals who were *CYP3A5* expressors (harboring CYP3A5*3 *AA or AG* genotype) than that in nonexpressors (CYP3A5*3 *GG genotype*) [34]. Besides, Qiu XY et al. observed in their study performed in 103 renal transplant recipients that the CYP3A4*1G AA genotype was found to have a lower dose-adjusted concentration [27]. The study was consistent with our results that the potentially higher metabolic capacity of CYP3A4*1G in patients with A allele in LT and we supposed that the association of recipients‘ CYP3A5*3 with Tac disposition was because of the gene linkage disequilibrium with recipients’ CYP3A4*1G partially.

Secondly, TBL was the third variable that independently predicted that Tac pharmacokinetics in addition to donors’ *CYP3A5* and recipients’ *CYP3A4* genotype. Plasma TBL was mainly related to heme-oxygenase (HO) which would influence the metabolism of heme consisted of hemoglobin [35], and in line with the strong binding of Tac to the red cell [36]. Besides, TBL could represent the function of the donor’s liver, where Tac was mostly metabolized. Notably, in our study, the selection of the study population might be responsible for the fact that no other biochemical or clinical variable predicted Tac pharmacokinetics. The choice of the Chinese population of adult liver transplant recipients who were tested 4 weeks after transplantation showed that some variables known to be associated with Tac disposition (i.e., ethnicity) could not affect Tac pharmacokinetics in our study. In addition, major drug–drug and drug-food interactions were avoided because the use of drugs and food that were known to either inhibit or induce *CYP3A* isoenzymes or to interfere with the absorption, distribution, metabolism, or excretion of Tac was not allowed, other than corticosteroids.

Thirdly, in the multivariate linear regression analysis, when the recipients’ CYP3A4*1G and donors’ CYP3A5*3 polymorphisms were combined, we found that the number of allele A, which combination of donors’ CYP3A5*3 and recipients’ CYP3A4*1G genotypes, correlated notably with Tac C/D variation at four weeks. Besides, extensive metabolizers, with the number of alleles A greater than or equal to two, showed lower dose-adjusted blood concentration than that of poor metabolizers with the number of alleles A less than two. Furthermore, the model of donors’ CYP3A5*3, recipients’ CYP3A4*1G, and TBL, for the prediction of Tac disposition was better than the model of donors’ CYP3A5*3 only at week 1, 2, and 3, the reasons for did not apparent at week 4 maybe were that (1) the concentration of Tac became steady at week 4 and the role of gene turned into small, especially CYP3A4*1G; (2) liver function presented by TBL transformed into normal. However, these views need to be confirmed in the further study with larger samples. We established a digital model to guide the clinically use of Tac; when the calculated *P* value was greater than or equal to 0.6685, the patients belonged to the category of a faster metabolism, which was consistent with our experimental results.

Fourthly, when combining the donors’ CYP3A5*3 and recipients’ CYP3A4*1G polymorphisms, there was a correlation between new-onset hypertension and the number of allele A, which in the combination of donors’ CYP3A5*3 genotype and recipients’ CYP3A4*1G genotype. If the number of alleles A more than two, the likelihood of new-onset hypertension was less. One of the reasons may be that allele A could affect the metabolism of Tac, which caused the development of hypertension by activating the renal sodium chloride co-transporter (NCC) [3], and the previous study also reported that Tac led to renal vasoconstriction and nephrotoxicity further confirmed our results [37, 38].

Overall, our results suggested that the donors’ CYP3A5*3, recipients’ CYP3A4*1G genotype, and TBL had a major influence on Tac exposure. Notably, to our knowledge, this was the first time to define individualized Tac doses in liver transplant patients according to our digital model consisted of genotypic CYP3A5*3, CYP3A4*1G, and clinical TBL, in a Chinese population. However, two limitations need to be acknowledged. Firstly, this study was based on small Chinese cohorts, most of them had hepatitis B virus-related liver diseases. Secondly, this was an observational study, and the basis of every important finding still required further explanation. In the future, a well-controlled clinical study was warranted to investigate this issue.

Furthermore, to date, this was the first study to explore the number of alleles A was associated with Tac disposition in LT in Chinese patients according to CYP3A5*3 and CYP3A4*1G combinations. This may allow the prevention of liver graft rejection and improve the safety profile of Tac. Besides, to our limited knowledge, this was the first time to define that the number of alleles A was associated with new-onset hypertension in liver transplant patients. This finding could be clinically relevant for the effective prevention, early diagnosis, and treatment of new –onset hypertension in Chinese LT recipients.

## MATERIALS AND METHODS

### Study subjects

Hundred patients from the China Liver Transplant Registry (CLTR) database who underwent orthotopic liver transplantation (OLT) from July 2007 to March 2012 at the First People's Hospital Affiliated to Shanghai Jiao Tong University were enrolled in this retrospective study. The exclusion criteria: (1) Patients with pre-existing hypertension or diabetes mellitus or hyperlipidemia before transplantation; (2) those who underwent re-transplantation or multiorgan transplantation; (3) those with less than 3 months of follow-up; (4) those who smoked or drank after operation were also excluded. The inclusion criteria of new-onset hypertension patients after transplantation according to China's organ transplant recipients hypertension guidelines and World Health Organization were:(1) systolic blood pressure>130mmHg and/or diastolic blood pressure>80mmHg, more than three consecutive measurements are taken, at least 5 minutes apart and with the person seated; (2) have antihypertensive drugs even though the blood pressure under the level above. The main causes of liver disease were hepatitis B virus (n=140). Lamivudine combined with low-dose intramuscular hepatitis B immunoglobulin therapy was applied in patients with hepatitis B virus-related liver disease. Amlodipine-based individualized antihypertensive treatment for recipients of hypertension. The immunosuppressive regimen was triple therapy incorporating Tac, mycophenolate, and steroid. After the initial study, a validating population of 70 patients from the CLTR database who underwent OLT between March 2012 and March 2015 at the First People's Hospital Affiliated to Shanghai Jiao Tong University was analyzed using the same methods of data collection, grouping, and genotyping as that of the training set.

### Ethics statement

Informed consent was obtained from all donors and recipients. Each organ donation or transplant was approved by the Institutional Review Board, First Hospital Affiliated Shanghai Jiao Tong University, strictly under the guidelines of the Ethics Committee of the hospital, the current regulation of the Chinese Government, and the Declaration of the Helsinki. No donor's livers were harvested from executed prisoners.

### Data collection

On the basis of previous studies, we used the Tac serum concentration to dose ratios (C/D ratio) for 28 days after transplantation as an index of Tac pharmacokinetics [32]. Trough blood concentration of Tac (ng/mL) was detected by PRO-Trac™ II Tac ELISA kit (DiaSorin Inc., USA) with microparticle enzyme immunoassay (ELx800NB analyzer, BioTek, USA). The daily dose (mg) of Tac was recorded, and the weight-adjusted dosage (mg/kg/d) was calculated. The Tac C/D ratio was calculated by dividing the Tac trough concentration (C0) by the corresponding weight-adjusted dosage. The results of the laboratory tests were also recorded. The average clinical data in the different periods were calculated to represent the corresponding clinical status.

### Genotyping

The liver tissue (20∼50 mg) was thawed from the donors and receptors, and the genomic DNA was extracted using the AllPrep DNA/RNA Mini Kit (Qiagen, Germany). Polymerase chain reaction (PCR) amplification was performed using a 2720 thermal cycler (Applied Biosystems). The primer sequences used were (a) *CYP3A5* *3: the forward primer: 5′- AGGAAGCCAGACTTTGATCATTATGTT-3′; the reverse primer: 5′- GAGAGTGGCATAGGAGATACCCA-3′ (b) CYP3A4*1G: the forward primer: 5′- ATGAACCAGAGCCAGCACGTTT-3′; the reverse primer: 5′- GCAGAAACTGCAGGAGGAAATTGAT-3′. The total volume of the PCR mix was 25 μL, and it contained 2.5 μL 10xPCR buffer, 50 pmol of each primer, 0.2 mM of each deoxynucleotide triphosphate, 2 U Taq DNA polymerase (TaKaRa Biotechnology Co., Ltd, Dalian, China), and 100 ng genomic DNA. The genotyping was carried out by direct sequencing on an ABI 3730 DNA Analyzer.

### Statistical analysis

The Kolmogorov-Smirnov test was used to check for normality. The Hardy–Weinberg equilibrium test was performed using an appropriate χ^2^ test. Pairwise r^2^ and D′ values for linkage disequilibrium were calculated using SHEsis software (http://analysis.bio-x.cn/myAnalysis.php). SPSS version 19.0 (SPSS Inc., Chicago, IL, USA) was used to complete other statistical analyses. Quantitative variables were expressed as mean ± SD and compared by Student’s t-test or Wilcoxon-Mann-Whitney test. Categorical variables were presented as values (percentages) and compared using Fisher’s exact test and Pearson’s χ^2^ test. Exploratory univariate correlation analysis (Spearman’s correlation coefficient) was performed to explore whether a specific covariate potentially affected the Tac pharmacokinetics. Tac does, Tac C0, and dose-corrected Tac C0 were used as dependent variables. All covariates that correlated with the Tac pharmacokinetic parameters at a *P* value < 0.2 in univariate correlation analysis were retained and entered into the multivariate linear regression model. The models were calculated in binary logistic regression, transformed in a new variable, and then compared using receiver operating characteristic (ROC) curve. According to the linear interpolation method, the false positive rate R = 10% was taken after points for diagnosis. In addition, categorical covariates were coded with a dummy variable set arbitrarily at 0 or 1 depending on the absence or presence of a specific feature. In a multivariate regression analysis, significant covariates of Tac pharmacokinetics were selected using the backward elimination procedure. A two-sided *P* < 0.05 was considered to be statistically significant.

## Abbreviations

Tac: tacrolimus
LT: liver transplantation
CLTR: China Liver Transplant Registry
TBL: total bilirubin
Hb: hemoglobin
C/D: dose-corrected Tac concentration
D/W: weight-corrected Tac dose
SNPs: single nucleotide polymorphisms
OLT: orthotopic liver transplantation (OLT)
ROC: receiver operating characteristic
AUC: area under the curve
BMI: body mass index
GPT: glutamate pyruvate transaminase
PCR: polymerase chain reaction
